# Hepatoprotective Activity of Methanolic Extract of *Bauhinia purpurea* Leaves against Paracetamol-Induced Hepatic Damage in Rats

**DOI:** 10.1155/2013/636580

**Published:** 2013-06-18

**Authors:** F. Yahya, S. S. Mamat, M. F. F. Kamarolzaman, A. A. Seyedan, K. F. Jakius, N. D. Mahmood, M. S. Shahril, Z. Suhaili, N. Mohtarrudin, D. Susanti, M. N. Somchit, L. K. Teh, M. Z. Salleh, Z. A. Zakaria

**Affiliations:** ^1^Department of Biomedical Science, Faculty of Medicine and Health Sciences, Universiti Putra Malaysia, 43400 Serdang, Selangor, Malaysia; ^2^Department of Animal Science, Faculty of Agriculture and Biotechnology, Universiti Sultan Zainal Abidin, Gong Badak Campus, 20300 Kuala Terengganu, Terengganu, Malaysia; ^3^Department of Pathology, Faculty of Medicine & Health Sciences, Universiti Putra Malaysia, 43400 Serdang, Selangor, Malaysia; ^4^Department of Biomedical Science, Kulliyyah of Science, International Islamic University Malaysia, Jl Sultan Ahmad Shah, Bandar Indera Mahkota, 25200 Kuantan, Pahang, Malaysia; ^5^Pharmacogenomics Centre (PROMISE), Faculty of Pharmacy, Universiti Teknologi MARA, 42300 Puncak Alam, Selangor, Malaysia

## Abstract

In an attempt to further establish the pharmacological properties of *Bauhinia purpurea* (Fabaceae), hepatoprotective potential of methanol extract of *B. purpurea* leaves (MEBP) was investigated using the paracetamol- (PCM-) induced liver toxicity in rats. Five groups of rats (n = 6) were used and administered orally once daily with 10% DMSO (negative control), 200 mg/kg silymarin (positive control), or MEBP (50, 250, and 500 mg/kg) for 7 days, followed by the hepatotoxicity induction using paracetamol (PCM). The blood samples and livers were collected and subjected to biochemical and microscopical analysis. The extract was also subjected to antioxidant study using the 2, 2-diphenyl-1-picrylhydrazyl (DPPH) radical scavenging assay with the total phenolic content (TPC) also determined. From the histological observation, lymphocyte infiltration and marked necrosis were observed in PCM-treated groups (negative control), whereas maintenance of the normal hepatic structural was observed in group pretreated with silymarin and MEBP. Hepatotoxic rats pretreated with silymarin or MEBP exhibited significant decrease (*P* < 0.05) in ALT and AST enzyme level. Moreover, the extract also exhibited antioxidant activity and contained high TPC. In conclusion, MEBP exerts potential hepatoprotective activity that could be partly attributed to its antioxidant activity and high phenolic content and thus warrants further investigation.

## 1. Introduction 

Diverse range of bioactive molecules has been isolated from plant natural product, making them medicinally valuable sources [1]. There has been a revival of interest in plant-based medicines due to the increase awareness of the limited ability of synthetic pharmaceutical products to control major disease and the need to discover new molecular structures as the lead compounds from other sources, including the plant kingdom [2]. One of the plants that are currently under investigation for its potential pharmacological activities in our laboratory is *Bauhinia purpurea *(family Leguminosae). Known to the Malays as “*pokok tapak kerbau,*” *B. purpurea* leaves have been traditionally used by the Indians to treat stomach tumors, ulcers, wounds, glandular swellings, diarrhea, and fever [3]. Scientifically, *B. purpurea* has been proved to possess antidiarrheal activity [4], thyroid stimulating and antihypothyroidism [5, 6], and larvicidal [7] activities. Other researchers have also reported the pharmacological benefit of *B. purpurea*. For example, the plant exhibited antimicrobial [8], antinociceptive, anti-inflammatory, and antipyretic [3, 9], antimycobacterial, antimalarial, antifungal, cytotoxic, and anti-inflammatory [10], anti-nephrotoxicity [11], and wound healing [12] activities. *In vitro* study has demonstrated that *B. purpurea *possesses antiproliferative [13], antioxidant [13, 14], and antimicrobial activities [14, 15], and also has potential as hepatocellular carcinoma inhibitor [16]. Interestingly, other studies have proved that* B. purpurea *leaf possesses antiulcer activity [17, 18]. We have also reported on the phytochemical constituents of *B. purpurea*, which indicate the presence of flavonoids, triterpenes, tannins, and steroids [3]. Flavonoids, in particular, are polyphenolic compounds, widely distributed in the plant kingdom, and exhibited various pharmacological activities including hepatoprotective activity [19]. Interestingly, there is a link between the hepatoprotective activity with the anti-inflammatory, antioxidation, and antiproliferative activities, which has been exerted by the leaves of *B. purpurea*. 

Our literature survey revealed that no attempt has been made to this date to study the hepatoprotective activity of *B. purpurea* leaves. Thus, we take this opportunity to study the hepatoprotective activity of methanol extract of *B. purpurea *leaves (MEBP) using the paracetamol- (PCM-) induced liver damage in rats as the animal model. 

## 2. Materials and Methods

### 2.1. Chemicals

Paracetamol (PCM; *Sigma*-Aldrich, USA) and silymarin (*Sigma*-Aldrich) were used in the present study. All other chemicals and reagents used were of analytical grade.

### 2.2. Collection of Plant Material

The leaves of *B. purpurea *were collected from their natural habitat around Universiti Putra Malaysia (UPM), Serdang campus, Selangor, Malaysia. A voucher specimen (SK 1985/11) was identified by comparison with specimens available at the Herbarium of the Laboratory of Natural Products, IBS, UPM, Serdang, Selangor, Malaysia. The leaves were dried under shade for 7 days at room temperature, segregated, and pulverized by mechanical grinder to form coarse powder. 

### 2.3. Preparation of Plant Extract

The coarse powder of *B. purpurea* had undergone the maceration type of extraction using methanol as the solvent system. The coarse powder of air-dried leaves of *B. purpurea* was subjected to methanol extraction whereby 1 kg of powder leaves was macerated in 20 L of methanol in the ratio of 1 : 20 (w/v) for 72 hours, and the supernatant was filtered sequentially using cloth filter, cotton wool, and Whatman no. 1 filter paper. The solvent was then evaporated under reduced pressure (204 mbar) and controlled temperature (40°C) using a vacuum rotary evaporator (Buchi Rotavapor R210/215, Switzerland). The residue was collected and subjected to the similar extraction process for another two times [20]. 

### 2.4. Pharmacological Studies

#### 2.4.1. Antioxidant Activity of MEBP

*Total Phenolic Content. *Determination of total phenolic content (TPC) was performed using Folin-Ciocalteu reagent according to the method of Singleton and Rossi [21] with slight modifications. Briefly, a 1.0 mg quantity of MEBP was extracted for 2 hours with 1.0 mL of 80% methanol containing 1.0% hydrochloric acid and 1.0% of distilled water at room temperature on the shaker set at 200 rpm. The hydrochloric acid was added at this stage as part of the extraction solution to increase solubility of insoluble plant compounds. The mixture was centrifuged at 6000 rpm for 15 minutes, and the supernatant decanted into vials. The supernatant was used for the determination of TPC. A 200.0 *μ*L of supernatant extract was mixed with 400.0 *μ*L of Folin-Ciocalteu reagent (0.1 mL/0.9 mL) and allowed to stand at room temperature for 5 minutes. Then, 400.0 *μ*L of sodium bicarbonate (60.0 mg/mL) solution was added, and the mixture was allowed to stand at room temperature for 90 minutes. Absorbance was measured at 725 nm. A calibration curve was generated by using the gallic acid standard optical density (OD), and the levels in the samples were expressed as gallic acid equivalent (GAE)-TPC mg/100 g. 

#### 2.4.2. DPPH Radical Scavenging Activity

Antioxidant reducing activity on DPPH radical was estimated according to the method of Blois [22] with modification involving the use of high-throughput microplate system. Sample (50 *μ*L of 1.0 mg/mL) was added to 50 *μ*L of DPPH (FG: 384.32) (1 mM in ethanolic solution) and 150 *μ*L of ethanol (absolute) in a 96-well microtiter plate in triplicates. The plate was shaken (15 seconds, 500 rpm) and left to stand at room temperature for 30 minutes. The absorbance of the resulting solution was measured spectrophotometrically at 520 nm.

### 2.5. Hepatoprotective Assay

#### 2.5.1. PCM-Induced Hepatotoxicity Test

The *in vivo* hepatoprotective activity of MEBP was determined using the PCM-induced hepatotoxicity test in rats. The animals were divided into 6 groups (*n* = 6) and administered with test solutions as described below.

- Group I served as normal control and received 10% DMSO
- Group II served as negative control and received 10% DMSO.
- Group III served as positive control and received 200 mg/kg silymarin.
- Pretreatment groups:
  1. Group IV received 50 mg/kg MEBP,
  2. Group V received 250 mg/kg MEBP, and
  3. Group VI received 500 mg/kg MEBP.

These doses of extract (50, 250, and 500 mg/kg) were used in the present study based on our previous reports on the acute toxicity study performed using the single dose of orally administered 5000 mg/kg MEBP, which showed no sign of toxicity in rats. Furthermore, this dose range was chosen based on the antiulcer activity of *B. purpurea* leaves [14]. Based on these findings, the highest dose used in the present study (500 mg/kg) was set up to be 10% of the dose used in the acute toxicity study (5000 mg/kg). In our preliminary study, the 500 mg/kg MEBP, which exhibited significant antiulcer activity, also exerted significant hepatoprotective activity. Therefore, the other two doses (50 and 250 mg/kg, which were 10 and 2 folds reduction of 500 mg/kg (the highest dose) were selected based on the antiulcer findings [14], respectively. 

The animals were fasted for 48 hours prior to the experiment under standard laboratory conditions. After 48 hours, each group of rats received the respective dose of test solution orally once daily for 7 consecutive days. The oral administration of PCM was performed 3 hours after the last extract administration on the 7th day except for group I, which received only 10% DMSO. Forty eight [23] hours after the hepatic injury induction, the animals were anesthetized using diethyl ether, and the blood was drained for biochemical parameters study. The animals were then sacrificed by cervical dislocation, and the liver was removed for histopathological studies. 

### 2.6. Biochemical Studies

Biochemical parameters were assayed according to the standard methods. Alanine aminotransferase (ALT), alkaline phosphate (ALP), aspartate aminotransferase (AST), total were measured using the Hitachi 902 Automatic Chemical Analyser. 

### 2.7. Histopathology

The liver tissue was dissected out and fixed in the 10% formalin, dehydrated in gradual ethanol (50–100%), cleared in xylene, and embedded in paraffin wax. The sections, which are 5-6 *μ*m thick, were then prepared using rotary microtome (Leica RM 2125 RTS, Singapore) and stained with hematoxylin and eosin dye for microscopic observation of histopathological changes in the liver. Liver sections then will be scored and evaluated according to the severity of the hepatic injury as described by El-Beshbishy et al. [24] with modifications. 

### 2.8. Phytochemical Screening and HPLC Analysis of MEBP

The phytochemical screening of MEBP was performed again according to the conventional protocols as adopted by Zakaria et al. [18]. The HPLC analysis of the extract was also carried out and performed according to the previous report [18] but with slight modifications. Briefly, 10 mg of MEBP was dissolved in 1 mL mETH and then filtered through the membrane filter (pore size 0.45 *μ*m). A Waters Delta 600 with 600 Controller and Waters 2996 Photodiode Array (Milford, MA, USA) equipped with an autosampler, online degasser, and column heater was used to analyse the filtered sample. Data were evaluated and processed using the installed Millenium 32 Software (Waters Product). The filtered samples were separated at 27°C on a minibore Phenomenex Luna 5 *μ*m C_18_ column (dimensions 250 × 4.60 mm) using a one-step linear gradient. The solvents were (A) 0.1% aqueous formic acid and (B) acetonitrile, and the elution system was as follows: initial conditions were 85% A and 15% B with a linear gradient reaching 25% B at *t* = 12 min. This was maintained for 10 min after which the programmed returned to the initial solvent composition at *t* = 25 min and continued for 10 min. The flow rate used was 1.0 mL/min, and the injection volume was 10 *μ*L. The HPLC was monitored at 254 and 366 nm.

### 2.9. Statistical Analysis

Data obtained are presented in mean ± standard error of mean (SEM). The data were analysed using the one-way analysis of variance (ANOVA), and the differences between the groups were determined using the Dunnett post hoc test as provided by the Graph pad PRISM V5.02 software. The limit of significance was set at *P* < 0.05.

## 3. Results 

### 3.1. *In Vitro* Antioxidant Studies

#### 3.1.1. Total Phenolic Compound

The result obtained showed that the total phenolic content of MEBP (200 *μ*g/mL) was 1194.35 ± 24.89 mg GAE/100 g. From the standard given, TPC value that higher than 1000 mg GAE/100 g is considered high total phenol compound. 

#### 3.1.2. DPPH Scavenging Assay

Free radicals scavenging capacity of MEBP was indicated by the bleaching of DPPH. Since the ability of MEBP to scavenge the free radical species in DPPH scavenging assay has been determined previously [13], the present study was carried out using only one concentration of the currently prepared MEBP with the aim of supporting the previous findings and, later, to propose the role of antioxidant effect as one of the possible mechanisms of hepatoprotective of MEBP. As can be seen from Table 1, the 200 *μ*g/mL MEBP caused more than 50% antioxidant activity when assessed against the DPPH radical scavenging assay, which is in comparison to the standard drug, 200 *μ*g/mL ascorbic acid.

### 3.2. *In Vivo* Hepatoprotective Study

#### 3.2.1. Effect of MEBP on the Body Weight and Liver Weight after Induction with PCM

PCM administration following pretreatment with 10% DMSO significantly (*P* < 0.05) caused augmentation in liver weight and body weight when compared to the normal control group. Interestingly, pre-treatment with 500 mg/kg significantly (*P* < 0.05) reduced the increased liver and body weight seen in the PCM-treated liver group. The reference hepatoprotective agent 200 mg/kg silymarin as expected has also demonstrated significant reduction in liver weight to normal value (Table 2). 

#### 3.2.2. Histopathological Study of the PCM-Induced Hepatotoxic Liver with and without Pretreatment with MEBP

**Figure 1:**
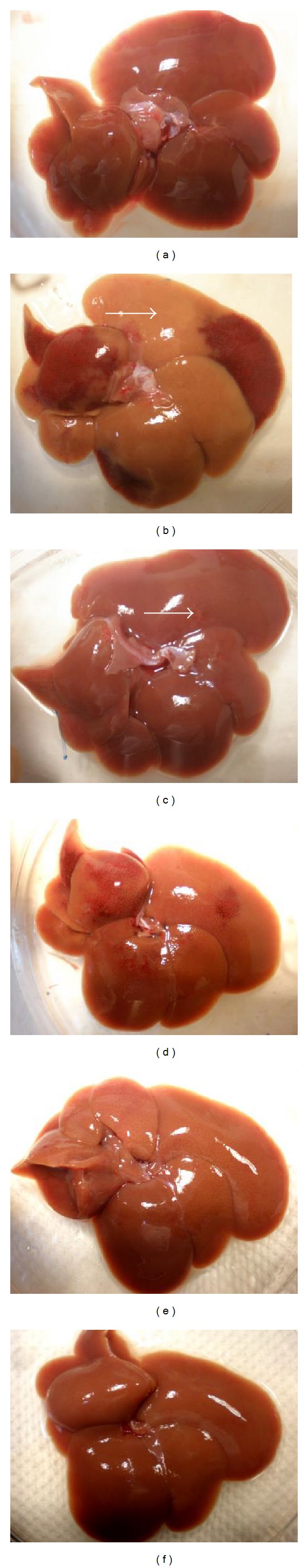
(a) Normal liver, (b) liver intoxicated with 3 g/kg PCM: gross image shows major color changes of liver lobes (arrow), (c) liver pretreated with 200 mg/kg silymarin and induced with PCM: spot of color changes was noted (arrow), (d) liver pretreated with 50 mg/kg MEBP and induced by PCM, (e) liver pretreated with 250 mg/kg MEBP and induced by PCM, (f) liver pretreated with 500 mg/kg MEBP and induced by PCM.

Gross necropsy and histopathological study were performed on the liver to observe any irregularities or abnormalities on the structure. Gross necropsy of normal liver demonstrated normal appearances (i.e., dark maroon in-color liver with smooth surfaces (Figure 1(a)). Meanwhile, the liver intoxicated with PCM showed major changes of the color of the lobes from maroon to brown (Figure 1(b)). Pre-treatment with 200 mg/kg silymarin (Figure 1(c)) or the MEBP (Figures 1(d)–1(f)) reversed the toxic effect of PCM with only mild spots of brown color changes observed. 

**Figure 2:**
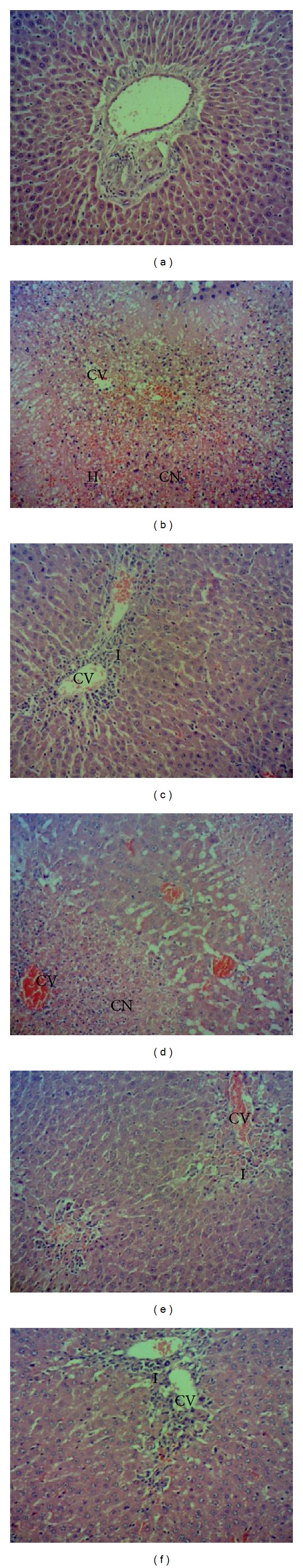
(a) Normal, (b) section of liver tissue of 3 g/kg PCM-treated group (p.o) showing massive coagulative necrosis, haemorrhage and inflammation. (c) Section of 200 mg/kg of silymarin liver tissue pretreated on the liver followed by PCM showing preservation of normal hepatocytes. (d) Section of pretreated 50 mg/kg MEBP liver tissue followed by PCM showing tissue necrosis and inflammation. (e) Section of pretreated 250 mg/kg MEBP liver tissue followed by PCM showing mild inflammation. (f) Section of pretreated 500 mg/kg MEBP liver tissue followed by PCM showing normal histology with mild inflammation. (100x magnification). CV: centrilobular. CN: coagulative necrosis. I: inflammation. H: haemorrhage.

Histopathologically, the non-PCM-intoxicated liver pretreated with 10% DMSO (normal) shows normal lobular architecture and normal hepatic cells with well preserved cytoplasm and well defined sinusoids line and nucleus around the perivenular area (Figure 2(a)). The section of PCM-intoxicated liver, pretreated with 10% DMSO, demonstrates infiltration of lymphocytes, the presence of haemorrhage and extensive coagulative necrosis of the perivenular, and midzonal region with periportal sparing (Figure 2(b)). Coagulative-type necrosis of hepatocytes in PCM-induced liver toxicity is present predominantly in the perivenular zone (zone 3). These pathological changes were found to be lesser as the dose of MEBP increased indicating the extract ability to reverse the PCM-induced intoxication (Figures 2(d)–2(f))).  Table 3 shows the histopathological scoring of the liver tissues pretreated with the respective test solution. Interestingly, the presence of marked necrosis, inflammation, and hemorrhage following treatment with PCM (shown by the negative control group) was reduced remarkably when pretreated with the extract or silymarin. 

#### 3.2.3. Biochemical Study

In this study, gross necropsy and histopathological study of the PCM-intoxicated liver pretreated with the respective test solution showed a correlation with serum biochemical indices. Paracetamol administration caused significant elevation in the ALT, AST, and ALP serum marker level in group pretreated with 10% DMSO as compared to the normal 10% DMSO-pretreated, non-PCM-intoxicated group (Table 4). However, oral administration of high dose MEBP (500 mg/kg) and silymarin (200 mg/kg) exhibited an ability to counteract the toxic effect of PCM by decreasing the level of these enzymes. 

### 3.3. Phytochemical Constituents and HPLC Profile of MEBP

The phytochemical screening of MEBP is shown in Table 5. Several groups of bioactive constituents were detected, namely, saponins, flavonoids, tannins and polyphenolic compounds, triterpenes, and steroids, but not alkaloids. However, the presences of those compounds are very low as indicated by the low froth formation (for detection of saponins) and weak colour formation (for detection of flavonoids, tannins, and triterpenes). 

**Figure 3:**
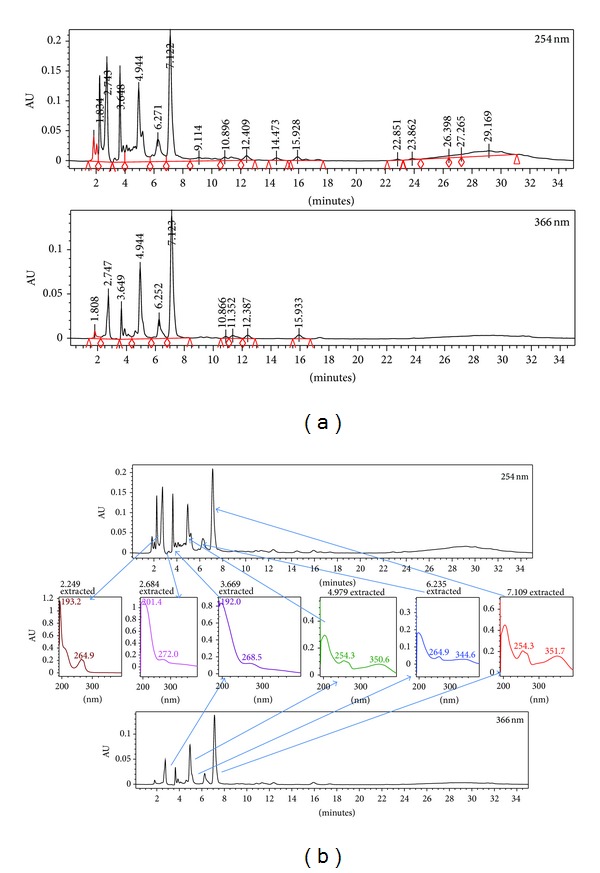
(a) The HPLC profile of MEBP at two different wavelengths, namely 254, and 366 nm. (b) The UV spectra analysis of peak 4 (RT = 4.94 min), peak 5 (RT = 6.27 min), and peak 6 (RT = 7.12 min) of the MEBP at 254 nm exhibiting the *λ* max at 254–351 nm, 264–345 nm,and 254–352 nm, respectively, suggesting, in part, the presence of flavonoid-based compounds.

The HPLC profile of MEBP measured at the wavelength of 254 and 366 nm is shown in Figure 2(a). The best wavelength wherein clear separation of peaks was obtained is 366 nm. At this wavelength, several peaks were separated with 5 major peaks (labelled as P1, P2 P3, P4, and P5) which were clearly detected in the chromatogram at the respective retention time (RT) of 2.75, 3.65, 4.94, 6.25, and 7.12 min, respectively. Further analysis demonstrated that the five peaks showed *λ*_max⁡_ values in the region of 201.4–272.0, 192.0–268.5, 254.3–350.6, 264.9–344.6, and 254.3–351.7 nm, respectively (Figure 3(b)).

## 4. Discussion and Conclusion

Hepatocytes are the main component that regulates various metabolic activities of liver. Distortion of this organ will result in disorder of body metabolism [25, 26]. An accidental over dosage administration of PCM as an antipyretic drug and over-the-counter analgesic [27] can result in hepatic damage [28]. *N*-acetyl-*p*-benzoquinoneimine (NAPQI), which is one of the metabolites of PCM after the latter undergoes metabolism in the liver via the action of cytochrome P450 (cyP450) monooxygenase [26, 29, 30], is highly responsible for the PCM toxic effect to the liver [31]. Several CYP450 enzymes have been known to participate in the bioactivation of NAPQI [30]. NAPQI is normally conjugated with glutathione (GSH) and excreted in urine. GSH has been highlighted to be responsible in the antioxidant defense of our body by scavenging the free radicals produced through the metabolism processes within the liver [32] in order to prevent any subsequent cell damage. Overdosage of PCM will result in accumulation of NAPQI, which will bind to GSH to form conjugates that will lead to the oxidation and conversion of GSH to glutathione disulfide (GSSG) resulting in the reduced level of blood and liver GSH [33]. Depletion of GSH level in blood and liver due to this process can result in mitochondrial dysfunction, increase of lipid peroxidation, and development of acute hepatic necrosis. Hepatocellular necrosis releases the enzymes such as AST and ALT into the circulation [34], and hence it can be measured in the serum [28]. Hepatic parenchymal cells produce pool of ALT that is regarded as specific enzyme for detection of liver abnormalities [35, 36]. Despite that, measurement of AST is still considered to be essential marker since it is sensitive to mitochondrial distortion predominantly in zone 3, centrilobular zone [35, 36]. Moreover, Somchit et al. [37] also suggested that NAPQI is involved in the formation of protein adducts via its action on DNA, proteins, cellular proteins, which in turn leads to the dysfunction and death of hepatocytes and finally liver necrosis. 

PCM-intoxication model is a universally established model to study the potential hepatoprotective activity of extracts/compounds [26, 38]. From the results obtained in the present study, PCM, at the intoxicated dose of 3 g/kg, increased the body weight and subsequently liver weight of rats as expected and showed significant elevation of serum level of hepatic enzymes, ALT and AST. Histopathological observations provide evidence of reducing number of viable cells with massive necrotic cells around the centrilobular zone extending to parenchymal zone, which is characterized by pyknosis and karyolysis nuclear. Interestingly, administration of MEBP showed capability to reduce the liver weight and concurrently caused significant reduction of enzyme ALT and AST level in blood in a dose-dependent manner. It is further supported by the normalization of histopathological changes to preserve the histostructure of hepatocytes. This liver regeneration progress was almost comparable to silymarin pretreated group which showed increasing number of viable cells and declining of hepatic enzymes level in serum.

 Based on the role of PCM metabolite, NAPQI, as described previously, the development of PCM-induced hepatotoxicity seems to depend partly on the existence of free radicals and oxidative processes. For that reason, it is hypothesized that extracts/compounds possessing free radical scavenging and/or antioxidant activities could also demonstrate hepatoprotective activity against the PCM toxic effect. This is supported by claim that the combination of hepatoprotective effect and antioxidant activity synergistically prevents the process of initiation and progress of hepatocellular damage [39]. Interestingly, our previous findings did demonstrate the MEBP ability to scavenge free radicals and to exert antioxidant activity [26], which is concurrent with our recent finding using the DPPH assay. Moreover, the inflammatory processes activated by PCM or other toxic agents are intimately involved in the chemical-induced hepatotoxic processes [40]. The inflammatory processes are thought to be responsible for producing various mediators, which are involved in the production of ROS and NO that can affect liver damage or repair. Therefore, it is also possible to postulate that extracts/compounds possessing anti-inflammatory activity might also exhibit hepatoprotective activity. It is again interesting to highlight that we have previously demonstrated that the leaves of *B. purpurea* possess anti-inflammatory activity [3, 9].

 Phytochemical screening of MEBP demonstrated the presence of flavonoids, saponins, condensed tannins, and steroids. In addition, the presence of high content of total phenolic compounds in the MEBP was reported in our previous and present study. The presence of flavonoids, in particular, has been confirmed earlier by Yadav and Bhadoria [41] followed by Zakaria et al. [18]. Flavonoids have been reported to exhibit antioxidant [42, 43], anti-inflammatory [44], and hepatoprotective [43, 44] activities. Furthermore, condensed tannins have been suggested to possess free radical scavenging and antioxidant, anti-inflammatory, and hepatoprotective activities [45], while saponins have been reported also to exhibit hepatoprotective activity via modulation of its antioxidant [46] and anti-inflammatory activities [47]. Taking all these reports into consideration, it is plausible to suggest that the hepatoprotective activity of MEMM involved, partly, synergistic action of flavonoids, condensed tannins, and saponins.

 Furthermore, the MEBP successfully reversed PCM-induced hepatotoxic effect, which is supported by the extract ability to bring down the elevated levels of ALT, AST and ALP, suggesting that these biochemical restorations could be due to the extract's inhibitory effects on cytochrome P450 or/and promotion of the PCM glucuronidation [48]. Moreover, the ability to bring down the enzymes level could be associated with the ability of MEBP to prevent peroxidative degradation of membrane lipids of endoplasmic reticulum that is rich in polyunsaturated fatty acids by thwarting binding of activated radicals to the macromolecules. This process could be achieved via the antioxidant activity of MEBP [23]. Other than that, several possible mechanisms could partly be linked to the observed hepatoprotective activity of MEBP. Mehendale [49] suggested that any potential mechanisms must involve activation of peroxisome proliferators activated receptor-*α* (PPAR-*α*), which are ligand-activated transcription factors activated by xenobiotics and are highly expressed in hepatocytes. Interestingly, Manautou et al. [50] and Liang et al. [51] have demonstrated the ability of flavonoids and saponins, which are also found in the MEBP, to activate the PPAR-*α* system. This mechanism of action might relate to the role of these compounds in the attenuation of PCM-induced hepatotoxicity. Interestingly, Han et al. [52] suggested that in the PCM-induced hepatotoxicity, regardless of which cellular pathway is involved in hepatoprotective activity, all pathways depend on PPAR-*α* receptor activation. In addition, mechanisms of hepatoprotection that could take place include activation of hepatic regeneration via an enhanced synthesis of protein and glycoprotein or accelerated detoxification and excretion [38], prevention of the process of lipid peroxidation, and stabilization of the hepatocellular membrane [23]. However, further studies are warranted before we could conclude on the exact mechanism(s) involved in the hepatoprotective activity of the MEBP such as cytokine assessments, antimitochondrial swelling activity, evaluation of oxidative stress markers, and immunohistochemistry study. 

## Figures and Tables

**Table 1:** Antioxidant profile of MEBP.

Sample	DPPH radical scavenging (%)
Ascorbic acid (200 *μ*g/mL)	85.72 ± 0.97
MEBP (200 *μ*g/mL)	61.06 ± 0.35

**Table 2:** Effect of MEBP on percentage change of body and liver weight in PCM-treated rats.

Treatment	Dose (mg/kg)	Change of body weight (%)	Liver weight (g/100 g)
Control	—	5.14 ± 0.43	2.89 ± 0.06
PCM		17.58 ± 2.10^a^	4.81 ± 0.40^a^
Silymarin + PCM	200	3.11 ± 0.67^b^	3.60 ± 0.11^b^
MEBP + PCM	50	15.57 ± 1.53^a^	5.66 ± 0.39^a^
	250	11.07 ± 0.73^ab^	4.29 ± 0.17^a^
	500	4.55 ± 0.91^b^	4.06 ± 0.24^ab^

Values are expressed as means ± S.E.M. of six replicates.

^a^Data differed significantly (*P* < 0.05) when compared to normal control within the respective column.

^b^Data differed significantly (*P* < 0.05) when compared to the negative control within the respective column.

**Table 3:** Histopathological evaluation of the effect of various doses of MEBP against PCM-induced hepatic injury in rats.

Treatment	Dose (mg/kg)	Steatosis	Necrosis	Inflammation	Haemorrhage
Normal	−	−	−	−	−
10% DMSO		−	+++	++	++
Silymarin	200	−	+	+	+
MEBP	50	−	++	+	+
	250	−	+	+	−
	500	−	+	+	+

The severity of various features of hepatic injury was evaluated based on those following scoring schemes: −: normal, +: mild effect, ++: moderate effect, +++: severe effect.

**Table 4:** Effect of MEBP pretreatment on ALT, AST and ALP (U/L) level.

Treatment	Dose (mg/kg)	ALT (U/L)	AST (U/L)	ALP (U/L)
Control	—	15.83 ± 2.862	95.13 ± 5.924	115.7 ± 6.994
PCM control (neg)		1714 ± 142.2^a^	3008 ± 210.7^a^	330.0 ± 42.35^a^
Silymarin (pos)	200	588.1 ± 193.7^ab^	959.2 ± 338.8^ab^	195.5 ± 11.06^ab^
MEBP	50	1222 ± 187.7^a^	2407 ± 294.9^a^	347.0 ± 29.40^a^
	250	1096 ± 221.1^ab^	2076 ± 409.4^ab^	264.8 ± 29.77^a^
	500	867.7 ± 101.2^ab^	1730 ± 256.6^ab^	256.3 ± 13.91^a^

Values are expressed as means ± S.E.M. of six replicates.

^a^Data differed significantly (*P* < 0.05) when compared to the normal control group within each respective column.

^b^Data differed significantly (*P* < 0.05) when compared to the negative control group within each respective column.

**Table 5:** Comparison on the phytochemical constituents between the leaves of *B.  pururea* and MEBP.

Sample	Phytochemical constituent	Result
MEBP	Alkaloid	—
	Saponin	+
	Flavonoid	+
	Tannins and polyphenolic compounds	+
	Triterpene	+
	Steroid	++

For saponins—+: 1-2 cm froth; ++: 2-3 cm froth; +++: >3 cm froth.

For flavonoids, tannins, triterpenes, and steroids—+: weak colour; ++: mild colour; +++: strong colour.

For akalioids—+: negligible amount of precipitate; ++: weak precipitate; +++: strong precipitate.
